# Exploring the Enzymatic and Antibacterial Activities of Novel Mycobacteriophage Lysin B Enzymes

**DOI:** 10.3390/ijms21093176

**Published:** 2020-04-30

**Authors:** Adel Abouhmad, Ahmed H. Korany, Carl Grey, Tarek Dishisha, Rajni Hatti-Kaul

**Affiliations:** 1 Division of Biotechnology, Department of Chemistry, Center for Chemistry and Chemical Engineering, Lund University, P.O. Box 124, SE-22100 Lund, Sweden; adel.abouhmad@biotek.lu.se (A.A.); carl.grey@biotek.lu.se (C.G.); 2 Department of Microbiology and Immunology, Faculty of Pharmacy, Al-Azhar University, Assiut 71524, Egypt; 3 Department of Microbiology and Immunology, Faculty of Pharmacy, Nahda University, Beni-Suef 62513, Egypt; ahmed.hassan@nub.edu.eg; 4 Department of Microbiology and Immunology, Faculty of Pharmacy, Beni-Suef University, Beni-Suef 62511, Egypt; Tarek.Dishisha@pharm.bsu.edu.eg

**Keywords:** mycobacteriophage, LysB, mycolylarabinogalactan esterase, mycolylarabinogalactan–peptidoglycan complex, antimycobacterial, lipolytic

## Abstract

Mycobacteriophages possess different sets of lytic enzymes for disruption of the complex cell envelope of the mycobacteria host cells and release of the viral progeny. Lysin B (LysB) enzymes are mycolylarabinogalactan esterases that cleave the ester bond between the arabinogalactan and mycolic acids in the mycolylarabinogalactan-peptidoglycan (mAGP) complex in the cell envelope of mycobacteria. In the present study, four LysB enzymes were produced recombinantly and characterized with respect to their enzymatic and antibacterial activities. Examination of the kinetic parameters for the hydrolysis of *para-*nitrophenyl ester substrates, shows LysB-His_6_ enzymes to be active against a range of substrates (C4–C16), with a catalytic preference towards *p-*nitrophenyl laurate (C12). With *p-*nitrophenyl butyrate as substrate, LysB-His_6_ enzymes showed highest activity at 37 °C. LysB-His_6_ enzymes also hydrolyzed different Tween substrates with highest activity against Tween 20 and 80. Metal ions like Ca^2+^ and Mn^2+^ enhanced the enzymatic activity of LysB-His_6_ enzymes, while transition metal ions like Zn^2+^ and Cu^2+^ inhibited the enzymatic activity. The mycolylarabinogalactan esterase activity of LysB-His_6_ enzymes against mAGP complex was confirmed by LC-MS. LysB-His_6_ enzymes showed marginal antibacterial activity when tested alone against *Mycobacterium smegmatis*, however a synergetic activity was noticed when combined with outer membrane permealizers. These results confirm that LysB enzymes are lipolytic enzymes with potential application as antimycobacterials.

## 1. Introduction

Mycobacterial infections remain among the deadliest and disabling diseases globally, the most common being tuberculosis (TB), a respiratory contagious disease caused by a direct contact with the acid-fast bacterium, *Mycobacterium tuberculosis* (*Mtb*). The increasing emergence of antibiotic resistance makes the disease a public health crisis [1]. In 2018, an estimated 10 million people developed TB, with half million rifampicin-resistant new cases (of which 78% had multi-drug resistant TB). Moreover, 1.2 million deaths were reported due to infection with *Mtb* in HIV-negative patients and 251,000 deaths among HIV-positive individuals [2]. Despite the existence of curative chemotherapy, the alarming rise of multidrug-resistant (MDR) and extremely drug-resistant (XDR) TB has increased the demand for developing novel and effective antimycobacterials [3,4].

The major characteristic feature of mycobacteria is the unique structure of their cell envelope with up to 60% lipid content compared to 5–10% for Gram-positive and -negative bacteria [5]. The cell envelope of Mtb consists of an inner peptidoglycan layer that is covalently linked to arabinogalactan, which in turn is esterified with mycolic acids (MAs). MAs are long chain (C60–C90), α-branched, β-hydroxy fatty acids containing cyclopropane rings, double bonds, and oxygenated groups according to the species and genera [6]. MAs are found in unbound and bound forms. The unbound form comprises glycolipid esters of trehalose forming trehalose dimycolate (TDM, also called the cord factor) that has a key role in mycobacterial pathogenesis [7,8]. The bound MA is linked via ester bond to the terminal pentaarabinofuranosyl units of arabinogalactan (AG), the polysaccharide that together with peptidoglycan forms the insoluble mycolylarabinogalactan–peptidoglycan (mAGP) skeleton [9,10,11,12,13,14]. Both forms of MAs participate in the two leaflets of the mycobacterial outer membrane, the mycomembrane [15,16], which imparts hydrophobicity and decreased permeability to nutrients and antimycobacterials making TB difficult to treat [17]. It is also essential for cell viability, hence the target of antituberculosis drugs [18].

Mycobacteriophages infecting the mycobacteria are able to disrupt the bacterial cell envelope from inside with the help of their lytic enzymes and release the viral progeny at the end of the lytic cycle. When applied externally mycobacteriophage-derived lysins can induce cell lysis and are thus considered as promising alternatives to conventional TB therapies [19]. Phage lysins have some advantages over the conventional antibiotics, for example they can counter drug-resistant pathogens [20,21,22], while presenting low risk of developing bacterial resistance [23], and can exert cell lysis even when the bacteria are in dormant state [23]. Mycobacteriophages produce two kinds of lytic enzymes, Lysin A (LysA) peptidoglycan hydrolases [24,25] and Lysin B (LysB) lipolytic enzymes [26,27]. LysB is a mycolylarabinogalactan esterase that cleaves the ester bond between arabinogalactan and MA, hence compromising the link between the mycobacteria cell wall and the outer membrane and completing the cell lysis [26,28]. LysB has also the ability to hydrolyze unbound mycolic acids (TDM) in different mycobacteria species including *Mtb* [26]. Gigante and coworkers evaluated the role of the mycobacteriophage M6 LysB (LysB-Ms6) in the cell lysis and reported that lysis can still occur after infection with the lysB deletion mutant (Δ*lysB*) of the mycobacteriophage, however the new Ms6 Δ*lysB* phage progeny is not efficiently released into the medium. Further cryo-electron microscopy and tomographic analyses at 150 min post-adsorption of the mutant phage revealed that *M. smegmatis* cells are incompletely lysed with the phage particles being trapped intracellularly, in contrast to complete cell lysis observed upon infection with the wild type mycobacteriophage M6 [29]. Also, a Δ*lysB* mutant of the mycobacteriophage Giles was shown to be defective in the normal timing, progression, and completion of host cell lysis [28].

To date, crystal structure of only one LysB, from mycobacteriophage D29, has been determined at 2.0Å resolution [28]. The structure revealed its similarity to α/β hydrolase superfamily of proteins including cutinases, lipases, and esterases. The enzymatic activity of LysB-D29 relies on the presence of the catalytic triad Ser82–Asp166–His240 with serine being part of the pentapeptide [G-X-S-X-G]. Recently, we have screened the Actinobacteriophage database (https://phagesdb.org/) with ≈1885 fully-sequenced mycobacteriophage genomes for the presence of putative LysB proteins through multiple sequence alignment and phylogenetic relationship with the amino acid sequence of the LysB-D29 [30]. The multiple sequence alignment showed Ser and Asp residues to be absolutely conserved in contrast to His which was weakly conserved through slight shifts either by one position towards the N-terminal end or the C-terminal end by 3 positions [28,30,31,32].

LysB candidates from 8 different mycobacteriophages (D29, Omega, Saal, Obama12, Enkosi, Echild, DS6A and Pumpkin) with homology ranging between 76 and 30% were selected for the present study, out of which only four LysB enzymes (LysB-D29, -Omega, -Saal and -Obama12) could be recombinantly produced in *Escherichia coli*. This paper presents the production and characterization of these LysB enzymes with respect to their enzymatic and antibacterial activities. The esterase activity using *p*-nitrophenyl esters, and lipase activity with Tween substrates were determined. The antibacterial activity of the enzymes was measured alone as well as in combination with anti-TB drugs (Rifampicin, Isoniazid, Pyrazinamide, and Ciprofloxacin), and with antimicrobial cationic polypeptides (Colistin and Protamine sulfate) against *Mycobacterium smegmatis,* a surrogate strain to the pathogenic *Mtb*.

## 2. Results

### 2.1. Cloning and Expression of LysB-His_6_ Genes

Cloning and transformation of the DNA sequences encoding LysB-His_6_ proteins (D29, Omega, Saal, Obama12, Enkosi, Echild, DS6A and Pumpkin) into *E. coli* BL21(DE3) resulted in successful expression of only 4 LysB proteins (D29, Omega, Obama12, and Saal) in soluble, active form in the recombinant bacteria grown in LB or auto-induction media. SDS-PAGE analysis of the enzymes revealed molecular weights consistent with predicted (https://web.expasy.org/protparam/) molecular masses of 29.3 kDa for LysB-D29, 31.5 kDa for LysB-Omega, 37.4 kDa for LysB-Saal, and 36.7 kDa for LysB-Obama12 with His_6_ tags (Supplementary Materials Figure S1). Expression of the other 4 LysB proteins (Enkosi, Echild, DS6A and Pumpkin) was not successful in spite of several attempts including different culture media, growth temperature post-induction, IPTG concentration, and even *E. coli* strains.

### 2.2. Activity of the LysB-His_6_ Enzymes Against p-Nitrophenyl Esters

Assay of the esterase activities of the recombinant enzymes against *p-*nitrophenyl esters, with different carbon chain lengths (C4–C18) showed that the enzymes exhibited varying trends in kinetic parameters against the different substrates (Table 1).

The highest activities (V_max_) for all the enzymes were obtained with the shortest ester *p*NPB (C4); the highest V_max_ recorded was 122.3 U/mg for LysB-D29 followed by the LysB-Omega (111.8 U/mg), while LysB-Saal and LysB-Obama12 exhibited significantly lower V_max_ values. There was a significant drop in V_max_ values on increasing the acyl chain length beyond C4, however increase in reaction rate to varying degrees was observed with *p*NPL (C12) for LysB-Omega, LysB-Saal, and LysB-Obama12, followed again by a decrease in V_max_. The trend in K_m_ values with the different substrates varied among the four enzymes, LysB-D29 exhibiting the lowest value with *p*NPL, LysB-Omega, and LysB-Obama12 with *p*NPP and LysB-Saal with *p*NPM. Interestingly, the highest catalytic efficiency (k_cat_/K_m_) for LysB-D29, LysB-Omega and LysB-Saal was observed with the C12 substrate, *p*NPL; the highest value being for LysB-Saal (4.31 µM^–1^. min^–1^). On the other hand, the highest k_cat_/K_m_ for LysB-Obama12 was 2.72 µM^–1^. min^–1^ with the C4 substrate *p*NPB. For all the LysB-His_6_ enzymes no activity was detected with *p*NPS (C18).

### 2.3. Activity of the LysB-His_6_ Enzymes Against Tween Substrates

**Figure 1:**
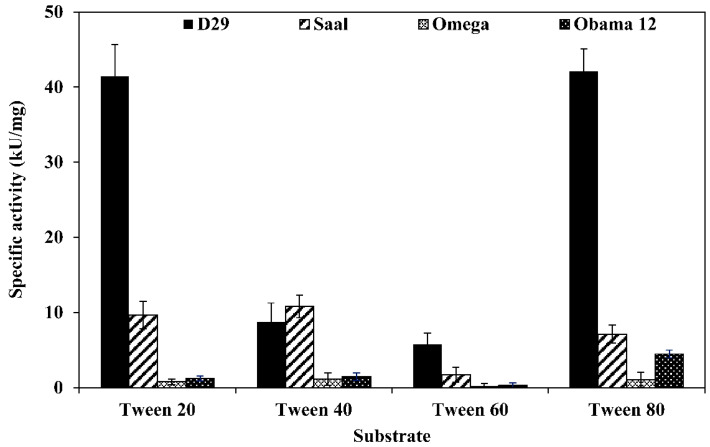
Specific activities of LysB-His_6_ enzymes against different Tween substrates. Fifty microliters of LysB-His_6_ solution (containing 0.175 µg of the enzyme) were mixed with 250 µL of the assay buffer (0.33% Tween, 50 mM Tris-HCl, 33 mM CaCl_2_, pH 8) and incubated at 30 °C for 1 h. The absorbance at 400 nm was recorded at 1 min intervals. One unit of lipase activity is defined as the amount of LysB-His_6_ enzyme that increases the optical density OD_400nm_ of 0.01/min under the assay conditions.

LysB-D29 was the most active enzyme against Tween substrates with prominent activities of 42.1 and 41.4 kU/mg against Tween 80 and 20, respectively. On the other hand, LysB-Saal exhibited highest activity against Tween 40 and 20 with activity values of 10.8 and 9.6 kU/mg, respectively, while LysB-Obama12 showed highest activity against Tween 80 (4.5 kU/mg) and lowest against Tween 60 (0.31 kU/mg). LysB-Omega exhibited relatively low activity against Tween substrates; the highest activity was 1.14 kU/mg against Tween 40 and 1.03 kU/mg against Tween 80 (Figure 1).

### 2.4. Data Correlation of Esterase and Lipase Activities

The correlation coefficients (−1 < *r* <1) for the esterase activity data sets against *p*-nitrophenyl esters and Tweens were calculated to determine if there is positive correlation (large values of one set are associated with large values of the other set) or negative correlation (large values of one set are associated with small values of the other set) or if there is no correlation at all between data sets. Table 2 shows the correlation coefficients displayed in the correlation matrix. Results for Tween 20, 60 and 80 hydrolysis exhibited strong correlation only with *p*NPB and *p*NPP, while Tween 40 showed strong correlation with all the tested *p*NP substrates.

### 2.5. LysB-His_6_ Activity Characteristics

**Figure 2:**
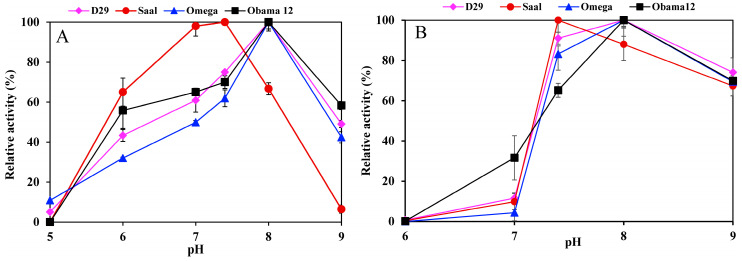
pH activity profile of LysB-His_6_ enzymes in pH range 5–9 using 20 mM phosphate buffer (pH 5–7) and 20 mM Tris–HCl (pH 7.4–9) against: (**A**) 1 mM *p*NPB at 37 °C (the 100% activity corresponded to 10, 9.77, 0.0524 and 0.0145 U/mg in case of *p*NPB for LysB-D29, –Omega, –Saal and –Obama12, respectively), (**B**) 0.33% Tween 80 at 30 °C (the 100% activity corresponded to 42.1, 1.058, 7.11 and 4.49 kU/mg in case of Tween 80 for LysB-D29, –Omega, –Saal and –Obama12, respectively). Symbols: LysB-D29 (♦), LysB-Saal (●), LysB-Omega (▲) and LysB-Obama12 (■). The experimental details are described in Section 4.4.1.

Determination of the esterase activity profiles of the LysB-His_6_ enzymes against *p-*nitrophenyl substrates in a pH range of 5–9 showed LysB-Omega, LysB-Saal and LysB-Obama12 to be optimally active at pH 8, and LysB-D29 at pH 7.4 (Figure 2A). Further increase in pH resulted in a sharp decrease in enzyme activities. The optimum pH for lipase activity against Tween substrates was 8 except for LysB-Saal that was optimally active at pH 7.4 (Figure 2B).

**Figure 3:**
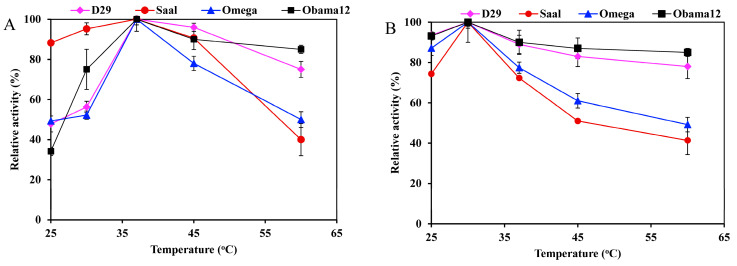
Temperature activity profiles of LysB-His_6_ enzymes against: (**A**) 1 mM *p*NPB, and (**B**) 0.33% Tween 80. The lipase activity assay was performed in a temperature range of 25–60 °C. Symbols: LysB-D29 (♦), LysB-Saal (●), LysB-Omega (▲) and LysB-Obama12 (■). The 100% activity corresponded to 10, 9.77, 0.0524 and 0.015 U/mg in case of *p*NPB, and 42.1, 1.058, 7.11, and 4.49 kU/mg in case of Tween 80 for LysB-D29, -Omega, -Saal, and -Obama12, respectively. The experimental details are described in Section 4.4.2.

The optimum temperature for the esterase activity against *p*NPB was 37 °C for all the enzymes; LysB-Obama12 and LysB-D29 retained over 80% activity at temperatures up to 60 °C (Figure 3A). On the other hand, the optimum temperature for lipase activity against Tween 80 was 30 °C, and 50% of the activity was lost at 60 °C (Figure 3B).

Effect of different additives including metal ions, EDTA and PMSF on the enzyme activities was also investigated. Mn^2+^ and Ca^2+^ ions stimulated the esterase activity of LysB-D29, -Omega and -Saal enzymes against *p*NPB but had an inhibitory effect on LysB-Obama12. Zn^2+^ and Cu^2+^ ions, on the other hand, decreased the activity of all the enzymes, and drastic inhibition of the activity was seen with phenylmethane sulfonyl fluoride (PMSF) (Table 3).

The metal ions (except for Mn^2+^) showed adverse effects on the lipase activity against Tween 80 of LysB-D29, Zn^2+^, and Cu^2+^ ions being the most inhibitory. In contrast, the lipase activity against Tween 80 of LysB-Omega, was boosted to 300%, 225%, and 212% of the original activity with Zn^2+^, Mn^2+^ and K^+^, respectively. Regarding LysB-Saal, Zn^2+^ totally abolished its activity, however EDTA enhanced the activity to 256% of original activity. The activity of LysB-Obama12 was stimulated by all the metal ions (except Zn^2+^ and Cu^2+^) as well as EDTA. The lipase activity of all LysB-His_6_ enzymes was inhibited by PMSF (Table 3).

### 2.6. Hydrolysis of mAGP

**Figure 4:**
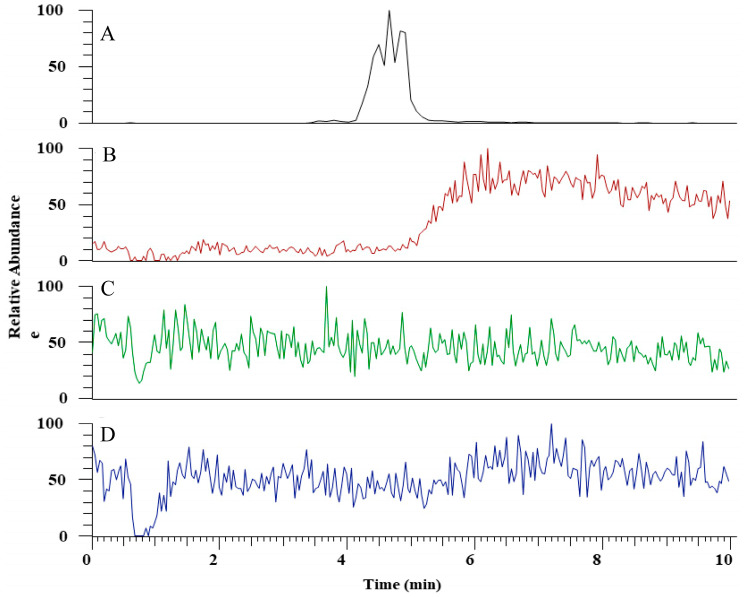
HPLC chromatogram for (**A**) mycolic acid standard, (**B**) mycolylarabinogalactan–peptidoglycan (mAGP) treated with LysB-D29, (**C**) mAGP treated with *Rhizopus oryzae* lipase, and (**D**) mAGP treated with buffer as negative control. The experimental details are described in Section 4.6.2.

**Figure 5:**
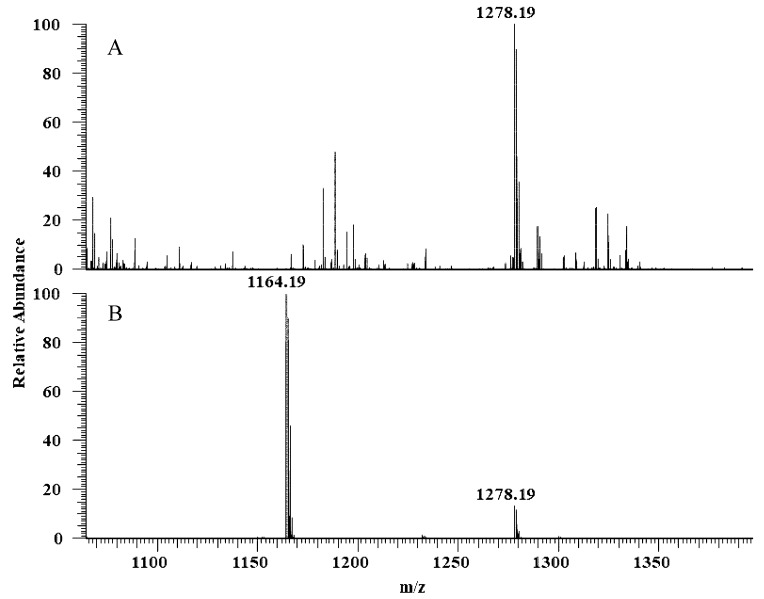
Full scan negative ions mass spectra of (**A**) mAGP treated with LysB-D29 with *m/z* 1278 corresponds to keto-mycolic acid, and (**B**) mycolic acid standard with *m/z* 1164 and 1278 corresponding to mycolic acid and keto-mycolic acid, respectively.

The hydrolytic activity of LysB-His_6_ enzymes against mAGP was evaluated using LC-MS in negative-ion mode. LysB attacks the ester bond between the arabinosyl units and mycolic acid, resulting in free mycolic acid. Figure 4 shows HPLC chromatogram for the different samples. mAGP treated with LysB-D29 reveals a peak corresponding to the liberated mycolic acid, while no peak was observed in the sample treated with *Rhizopus oryzae* lipase and the negative control. The observed m/z value of 1278.19 confirmed the presence of mycolic acid in LysB-D29 treated mAGP samples (Figure 5).

### 2.7. Antibacterial Activity

LysB-His_6_ enzymes did not show any antibacterial activity against *M. smegmatis* as a function of MIC and MBC, when tested in different media. However, a marginal Log_10_ reduction of 1.1, 1.32, 1.44, and 1.36 for LysB-D29, -Omega, -Saal, and -Obama12, respectively could be detected with 100 µg/mL of LysB-His_6_ in Cation Adjusted Mueller-Hinton Broth (CAMHB) (Table 4). Moreover, combining LysB-His_6_ enzymes with different anti-TB drugs did not exert synergistic effect. On the other hand, the inhibitory effect of LysB-His_6_ enzymes was enhanced by addition of half MIC values of Colistin and Protamine sulfate, respectively; the highest reduction being obtained with Obama12 (Table 4 and Supplementary Materials Table S1). Incubation of *M. smegmatis* cells with Colistin and Protamine sulfate at half MIC values did not result in a decrease in the Log_10_ values.

### 2.8. Thermostability of LysB-His_6_ Enzymes

Thermostability of the enzymes measured as the melting temperature (T_m_) using Nano DSF, showed LysB-Omega to be the most stable enzyme with a T_m_ value of 57.7 °C followed by LysB-D29 (T_m_ of 54.7 °C). On the other hand, LysB-Obama12 and -Saal showed lower T_m_ values of 47.9 °C and 45.7 °C, respectively (Table 5).

## 3. Discussion

Despite the availability of ≈1885 mycobacteriophage complete genome sequences available on the Actinobacteriophage database (https://phagesdb.org/) (accessed April 2020), the knowledge of their lytic enzymes is fairly limited. Concerning LysB enzymes, crystal structure of only LysB-D29 is available so far and some studies on the antimycobacterial properties of a few other LysB candidates have been reported [26,27,28,32,33]. The present study was started by selection of 8 LysB candidates which exhibited 30–76% sequence analogy with LysB-D29. Only 4 (including LysB-D29) of the 8 LysB enzymes could be expressed and purified to homogeneity. Payne and coworkers have earlier observed high variations in expression and solubility levels of LysB proteins and were able to obtain good expression levels of only LysB-D29 among trials with expression of several LysB enzymes [28].

LysB enzymes hydrolyze the ester bonds between the mycolic acid and arabinogalactan as well as the disaccharide trehalose. The three LysB enzymes characterized so far have exhibited structural relatedness and activity patterns similar to the lipolytic enzymes belonging to the α/β hydrolase family including esterases, lipases, and cutinases [32]. While esterases catalyze the hydrolysis of glycerol esters with short acyl chains and lipases act on long chain triacylglycerol substrates [34], cutinases act on wide range of substrates from soluble *p-*nitrophenyl esters to insoluble long-chain triglyceride esters besides its natural substrate cutin [35,36]. LysB enzymes are considered to be a link between lipases and cutinases as they have the ability to hydrolyze soluble esters and emulsified triglycerides [27]. As reported earlier, amino acid sequence analysis revealed the cutinase motif to be conserved among the majority of LysB enzymes [30,31]. Our recent study has further reported several common structural features of Lys B enzymes with the α/β hydrolase members [30].

The lipolytic activities i.e., esterase and lipase activities of LysB-His_6_ enzymes were evaluated against *p*-nitrophenyl esters and Tween substrates, respectively, both with varying carbon chain lengths. In general, the activity levels of the different enzymes varied significantly with respect to the different substrates. Measurement of the esterase activity against *p*-nitrophenyl esters (C4–C16) showed 3 of the enzymes to exhibit highest catalytic efficiency towards C12 (*p*NPL) (Table 1), which is in accordance with the earlier report on LysB-Ms6 having the highest catalytic efficiency of 1.14 µM^–1^. min^–1^ against *p*NPL [27]. The decrease in the esterase activity of LysB-D29 and other LysB enzymes with increasing chain lengths of the *p*-nitrophenyl ester substrates is also in agreement with the previous report on LysB-D29 showing the highest activity against the C4 substrate *p*NPB (0.72 U/mg) [28]. It was further reported that the K_m_ values of LysB-Ms6 and -Bxz2 decreased with the increase in carbon chain length of the *p*NP esters [33]. LysB-Bxz2 showed 10-fold higher esterase activity than Ms6 but with lower catalytic efficiency [33]. This might be attributed to the different conformations of the active sites of LysB proteins (tunnel, shallow bowl, deep, superficial funnels and deep buried cave) influencing the binding of different substrates as suggested in our previous study [30]. We noted that LysB enzymes with longer hydrophobic acyl binding site, e.g., LysB-D29 (tunnel) and LysB-Omega (shallow bowl) have higher V_max_ values against long fatty substrates than the LysB enzymes with shorter ones, e.g., LysB-Saal (deep funnel) and LysB-Obama12 (inverted tunnel).

Tunnel-shaped active sites of LysB-D29 and -Obama12 confer long, open hydrophobic cervix that accept long chain fatty substrates (Supplementary Materials Figure S3a). However, the inverted positioning of *p*NP ligands upon docking to LysB-Obama12 active site (Supplementary Materials Figure S3b) directs the long hydrophobic ligand tail towards the narrow hydrophilic mouth of the tunnel where no sufficient hydrophobic acyl binding site is provided. This might explain the highest activity of LysB-D29 on *p*NPP (C16) among the tested LysB-His_6_ enzymes in contrast to LysB-Obama12 with the lowest activity against the same substrate (Table 1). Furthermore, the shallow bowl conformation, was shown earlier to accept long substrates up to *p*NPS with C18 acyl chain length [37]; it provides a long hydrophobic superficial groove for fitting the long hydrophobic tail of *p*NP ligands, which might account for LysB-Omega achieving the second highest specific activity on *p*NPP (Supplementary Materials Figure S3c). On the other hand, LysB-Saal has a deep funnel conformation, which was reported earlier to suit bulky ligands with acyl chain length up to C14, with a narrow opposing hydrophobic wall wrapping the fatty *p*NP tail (Supplementary Materials Figure S3d). The narrow and short acyl binding site of LysB-Saal might explain its diminished activity against fatty substrates with long acyl side chain and its highest affinity towards *p*NPM (Table 1) [30]. In contrast, shorter substrates (up to C8) are better accepted by shorter and shallower funnel conformation e.g., LysB-Bxz2 [30,31,37].

Tweens 20, 40, 60, and 80 are esters of lauric (C12), palmitic (C16), stearic (C18), and oleic (C18) acids. Tween 20 and 80 turned out to be the best substrates for most of the enzymes, probably due to the relatively lower melting point of the corresponding fatty acids, especially for oleic acid. However, LysB-Saal showed higher activity against Tween 40 (10.8 kU/mg) (Figure 1). The higher enzymatic activity recorded against Tweens compared with *p*-nitrophenyl substrates may be attributed to the high emulsifying properties of the detergents forming stable oil-in-water emulsions, allowing for greater interactions between the substrate and the enzyme [38,39]. It is worthy to note that the mycobacterial cutinases as well as phospholipase A isolated from *M. smegmatis* show the same patterns of activity correlation between Tweens and *p*NP substrates [40,41,42,43].

LysB-His_6_ enzymes were shown to be active over a wide range of temperature (with optimum of 37 °C against *p*NPB and 30 °C against Tween 80) and in the pH range of 7.4–8, which seems to be similar to the activity pattern of different lipolytic enzymes reported earlier [44]. The melting temperatures of LysB-His_6_ enzymes ranged between 45–60 °C which correlates well with both lipase and esterase activities of the enzymes; increasing the reaction temperature above 60 °C, resulted in drop of the enzymatic activity of more than 60% for some of the enzymes. As reported earlier for LysB-Ms6 [27], the metal ions Mn^2+^, Ca^2+^, and Mg^2+^ generally showed a stimulating effect on the esterase and lipase activities of most LysB-His_6_ enzymes, while the transition metal ions, Zn^2+^ and Cu^2+^ had an inhibitory effect (Table 3). Activation by Ca^2+^ and inhibition by Zn^2+^ and Cu^2+^ have also been reported for esterase/lipase from *Geobacillus thermoleovorans* [45]. The effect of Ca^2+^ is related to the structural stabilization and activation of lipases [46,47], while Zn^2+^ and Cu^2+^ ions lead to activity loss due to the metal ion catalyzed oxidation of the sensitive amino acids such as histidine that is an important part of the catalytic triad in the α/β hydrolases [48,49]. EDTA had a slight inhibitory effect on the esterase activity that may suggest chelation of some stimulatory metal ions present in the samples [50]. On the other hand, the reason underlying the significant increase in the lipase activity of most of the enzymes with EDTA is not entirely clear but may be related either to increased flexibility of the enzyme or complexation of potential inhibitory compounds in the reaction. The activity inhibition by PMSF is attributed to its interaction with the conserved nucleophilic Ser residue in the catalytic triad, which is typical of esterases, lipases as well as proteases, hence confirming that LysB enzymes belong to the group of serine hydrolases [27,51].

The hydrolytic activity of LysB-His_6_ enzymes against the isolated mAGP substrate is also in accordance with the previously reported mycolylarabinogalactan esterase activity of other LysB homologous enzymes Ms6 [26], Bxz2 [33], D29 [28], and Bxb1 [52]. This confirms that the LysB enzymes are capable of hydrolyzing the ester bonds not only from inside the cells when viral progeny is released but also when applied from outside.

Earlier investigations on LysB-Ms6, -Bxz2 [33], LysA-D29 [53], BTCU-1 LysA and B [54], LysA-Ms6 [55], and LysA-TM4 [56] have revealed no significant antibacterial activity against *M. smegmatis* except for a combination of BTCU-1 LysA and BTCU-1 LysB that showed higher antibacterial activity (minimum bactericidal concentration (MBC) of 20–40 µg/mL) than LysA alone (MBC > 80 µg/mL) against *M. smegmatis*. Although the mechanism is not entirely clear, the synergistic effect is most likely due to the ability of the enzymes to penetrate through the peptidoglycan layer and disrupt the integrity of the mycolic acid linkage to the arabinogalactan-peptidoglycan layer.

LysB-His_6_ enzymes in this study did not exhibit significant antibacterial activity either alone or in combination with anti-TB drugs. The activity was determined without Tween 80, which has been suggested to be required for removal of *M. smegmatis* cell clumps and aggregates due to surface hydrophobicity and also for promoting the antibacterial activity of LysB enzymes [33,57,58,59,60]. There is also a possibility that the antibacterial activity observed in the presence of Tween 80 could be due to the action of the oleic acid released by hydrolysis of the surfactant by LysB [26,33]. The differences in the antibacterial activity among the LysB-His_6_ enzymes tested in this study are very subtle when compared with the high diversity of their catalytic profiles (in terms of esterase and lipase activities), which is clearly related to the diversity of active site conformations among LysB enzymes.

While LysB-His_6_ enzymes did not exert notable inhibitory effect against *M. smegmatis* cells, half MIC values of the polycationic peptides colistin and protamine sulfate enhanced the Log_10_ reduction of *M. smegmatis* treated with 100 µg/mL LysB-His_6_ enzymes (Table 5). Colistin and Protamine sulfate are cyclic cationic polypeptides with a hydrophobic tail, which destabilize the bacterial outer membrane by quenching the divalent cations (Ca^2+^ and Mg^2+^) from the lipopolysaccharides and phospholipids creating pores and subsequently cell death [61]. The antibacterial activity of cationic polypeptides extends beyond Gram-negative bacteria to different species of Mycobacteria including *M. avium* [62], *M. aurum*, *M. vaccae* [63], *M. xenopi* [64], *M. smegmatis* [65], and *Mtb* [60,65]. Due to the permeabilizing effect of Colistin on Mycobacteria cell membrane, it has been used in combination with anti-TB drugs to facilitate their uptake [66,67].

Even with the combination of LysB-His_6_ and polycationic peptides, we could not detect MIC/MBC values. For achieving significant antimycobacterial activity, a combination of antimicrobial peptides (outer membrane permeabilizer), LysB (mAGP hydrolase) and LysA (peptidoglycan hydrolase) should be considered. Earlier reports have demonstrated that fusions of antimicrobial peptide with either LysA or LysB did not exert antimycobacterial activity, however mixing the two fusion proteins together resulted in enhanced antimycobacterial activity even for intracellular mycobacteria in lung macrophages cell line [68].

## 4. Materials and Methods

### 4.1. Bacterial Strains, Vectors, Media, and Reagents

The genes encoding LysB enzymes from different mycobacteriophages (D29, Omega, Obama12, Enkosi, Echild, DS6A, Pumpkin and Saal) were codon optimized, fused with a hexa-histidine tag (His_6_) at the C-terminus (Supplementary Materials Figure S2) and ordered as gBlock gene fragments from Integrated DNA Technologies (IDT, Leuven, Belgium). *Escherichia coli* BL21(DE3) expression host and pET22b(+) expression vector were purchased from Novagen (Madison, WI, USA). Luria–Bertani (LB) medium was obtained from Saveen & Werner AB (Limhamn, Sweden). Isopropyl β-D-1-thiogalactopyranoside (IPTG), *EcoR*I, *Nde*I and T4 DNA ligase were products of Thermo Fisher Scientific (Waltham, MA). Ampicillin, *p-*nitrophenyl butyrate (*p*NPB; C4), *p-*nitrophenyl octanoate (*p*NPO; C8), *p*-nitrophenyl laurate (*p*NPL; C12), *p*-nitrophenyl myristate (*p*NPM; C14), *p*-nitrophenyl palmitate (*p*NPP; C16) and *p*-nitrophenyl stearate (*p*NPS; C18) were procured from Sigma-Aldrich (St Louis, MO, USA).

*M. smegmatis* mc^2^ 155 (ATCC 700084) was procured from American Type Culture Collection (ATCC) (Manassas, VA, USA) and grown in Middlebrook 7H9 broth medium (Difco, Detroit, MI, USA) supplemented with 0.2% glycerol, 0.05% Tween 80 and ADC (Albumin-Dextrose Complex) at 37 °C, 200 rpm. Glycerol stocks of *M. smegmatis* were prepared and stored at −80 °C. The bacteria were also grown on Middlebrook 7H10 agar medium supplemented with OADC (Oleic acid-Albumin-Dextrose Complex), 0.5% glycerol and 0.05% Tween 80.

### 4.2. Cloning and Expression of LysB-His_6_ Genes

Cloning and expression of LysB-His_6_ gBlocks were performed as reported earlier [30]. This involved cloning in *EcoR*I and *Nde*I restriction sites of pET22b(+) expression vector, ligation with T4 DNA ligase, and transformation of the ligation mixtures into *E. coli* BL21(DE3) expression host. The bacteria were grown overnight at 37 °C on LB agar plates supplemented with 100 µg/mL ampicillin. The plasmids (pET22b(+)-LysB-His_6_) extracted from the transformant colonies were sequenced (GATC Biotech AB, Solna, Sweden), and those with correct sequences were used for protein expression in *E. coli* BL21(DE3), which was grown overnight at 37 °C, 200 rpm in LB medium supplemented with the antibiotic as above. The recombinant cells were suspended in 50% glycerol, distributed in xx μL aliquots and stored at −80 °C.

Small scale production of the recombinant LysB-His_6_ enzymes was done in LB medium supplemented with 100 μg/mL ampicillin. The respective glycerol stocks were inoculated into 10 mL of the medium in 50 mL sterile falcon tubes and grown overnight at 37 °C and 200 rpm, and 5 mL of the culture were used to inoculate 50 mL medium in 250 mL Erlenmeyer flask and grown under the same conditions. When the optical density (OD600_nm_) reached 0.5−0.6, the cells were induced for protein expression using IPTG at a final concentration of 1mM and reducing the cultivation temperature to 30 °C. After 4 h, the cells were harvested by centrifugation at 3900× *g* and 4 °C for 15 min (Sigma 3−16PK, Sigma Laborzentrifugen GmbH, Osterode am Harz, Germany).

Protein production at a larger scale was performed in auto-induction medium (1% tryptone, 0.5%, yeast extract, 25 mM Na_2_HPO_4_, 25 mM KH_2_PO_4_, 25 mM (NH_4_)_2_SO_4_, 2 mM MgSO_4_, 0.05% glucose and 0.2% α-lactose) supplemented with 100 µg/mL ampicillin. One liter of the auto-induction medium in 5 L Erlenmeyer flask was inoculated with 15 mL of recombinant cell culture grown in LB medium as above, incubated at 37 °C and 180 rpm for 4 h, and then at 30 °C for 24 h prior to harvesting the cells by centrifugation at 6000× *g* and 4 °C for 20 min (Sorvall Lynx 4000 centrifuge, Thermo Scientific, Waltham, MA, USA).

### 4.3. Purification of LysB-His_6_ Enzymes

The cell pellet obtained above from 1 L auto-induction medium was suspended in 50 mM Tris-HCl buffer, pH 8 supplemented with 50 mM NaCl and a cocktail of protease inhibitors (Calbiochem) and then sonicated on ice (5 × 60 s, cycle 0.5) using UP400S sonicator (Dr. Hielscher GmbH, Stahnsdorf, Teltow, Germany). After removal of the cell debris by centrifugation (18,500× *g*, 30 min, 4 °C, Sorvall RC5C, Sorvall Instruments, Dupont, Wilmington, DE, USA), the clarified lysate was subjected to immobilized metal ion affinity chromatography (IMAC) for purification of the LysB-His_6_ enzymes using 5 mL HisTrap FF^TM^ nickel column (GE Healthcare Bio-Sciences AB, Uppsala, Sweden). Briefly, 15 mL of the clarified lysate was applied on the column pre-equilibrated with the binding buffer (50 mM Tris-HCl, 0.3 M NaCl, 20 mM imidazole, pH 8), the unbound proteins were washed out using a wash buffer (50 mM Tris-HCl, 0.3 M NaCl, 40 mM imidazole, pH 8), and finally the bound proteins were eluted with the elution buffer (50 mM Tris-HCl, 0.3 M NaCl, 0.5 M imidazole, pH 8). The purified proteins were dialyzed against dialysis buffer (50 mM Tris-HCl, 50 mM NaCl, 50% glycerol, pH 8), analyzed by SDS-PAGE and quantified with bicinchoninic acid (BCA) reagent using bovine serum albumin (BSA) as a standard (Sigma-Aldrich, St Louis, MO, USA), prior to storage at −20 °C.

### 4.4. Activity Assays of LysB-His_6_ Enzymes

#### 4.4.1. Activity Against *p*-Nitrophenyl Esters

The esterase activity of the purified LysB-His_6_ enzymes was measured against *p*-nitrophenyl esters with variable carbon chain length as substrates. Stock solutions (250 mM) of *p*NP-esters were prepared in dichloromethane and stored at −20 °C. For the assay, 180 µL of *p*NP-esters (final concentration of 1 mM dissolved in 20 mM Tris-HCl, 100 mM NaCl, 0.1% Triton X-100, pH 8) was mixed with 20 µL LysB-His_6_ enzyme solution (containing 0.175 µg enzyme). The reaction mixture was incubated at 37 °C and the release of *p-*nitrophenol was followed at 410 nm with 1 min intervals for 30 min (Multiskan^TM^ GO Microplate Spectrophotometer, Thermo Scientific, Vantaa, Finland). Each assay was performed in three independent replicates, using the corresponding buffer as negative control unless otherwise stated.

The activity was calculated and quantified with the following equation using calibration curve for *p-*nitrophenol.

(1) $$\frac{\mathrm{Units}}{\mathrm{mL}}\mathrm{enzyme}\;=\frac{\left(\Delta A410\;\mathrm{test}–\Delta A410\;\mathrm{blank}\right)\times \left(\mathrm{Total}\;\mathrm{reaction}\;\mathrm{volume}\right)}{\varepsilon \;\times \left(\mathrm{Volume}\;\mathrm{of}\;\mathrm{enzyme}\;\mathrm{in}\;\mathrm{the}\;\mathrm{reaction}\right)}$$

where ε is the extinction coefficient of *p*-nitrophenol which was determined under the assay conditions and at different pH values. One unit (U) of enzyme activity corresponds to the amount of enzyme liberating 1 µmol of *p*-nitrophenol per min under the assay conditions.

Optimum conditions for the enzyme activity were determined using 1 mM *p*NPB as substrate. To determine the optimum temperature, the assay was performed in a temperature range of 25–60 °C, while optimum pH was determined in a pH range of 5–9 using 20 mM sodium phosphate buffer (pH 5–7) and 20 mM Tris-HCl (pH 7.4–9), respectively.

The substrate specificity of the LysB-His_6_ enzymes was measured using *p*NP esters with variable carbon chain length ranging from C4 to C18. The kinetic parameters were determined by running the enzymatic activity assay using the substrates in a concentration range of 10 µM–4 mM and calculated according to the Michaelis-Menten model.

The effect of metal ions (5 mM Ca^2+^, Mn^2+^, Cu^2+^, Mg^2+^, Zn^2+^, K^+^, Na^+^), 5 mM EDTA and 10 mM phenylmethane sulfonyl fluoride (PMSF), respectively, was determined by incubation with LysB-His_6_ enzymes at room temperature for 2 h, and then determining the residual esterase activity using *p*NPB as substrate under the standard assay conditions at pH8 and 37 °C. The relative activity (%) was calculated in relation to the activity of the enzymes incubated under the same conditions but without any metal ions.

(2) $$\%\mathrm{Relative}\;\mathrm{activity}\;=\frac{\left(\mathrm{Enzymatic}\;\mathrm{activity}\;\mathrm{with}\;\mathrm{metal}\;\mathrm{ion}\right)\times 100}{\mathrm{Enzymatic}\;\mathrm{activity}\;\mathrm{without}\;\mathrm{metal}\;\mathrm{ion}}$$

#### 4.4.2. Activity Against Tween Substrates

The lipase activity of LysB-His_6_ enzymes was determined against Tween 20, 40, 60, and 80 as substrates [32], which are esters of lauric, palmitic, stearic, and oleic acids, respectively. The assay is based on the cleavage of Tween into alcohol and the corresponding fatty acids, which in the presence of calcium ions form insoluble calcium salts of fatty acids that can be measured turbidimetrically at 400 nm. In a microtiter plate, 250 µL of the assay buffer (composed of 0.33% Tween dissolved in 50 mM Tris-HCl, 33 mM CaCl_2_, pH 8) was mixed with 50 µL of LysB-His_6_ enzymes (final amount of 0.175 µg in the reaction mixture) and incubated at 30 °C for 1 h. The absorbance at 400 nm was recorded at 1 min intervals (Multiskan^TM^ GO Microplate Spectrophotometer). One unit of lipase activity is defined as the amount of LysB-His_6_ enzyme that increases the optical density OD400_nm_ of 0.01/min under the assay conditions.

Optimum temperature, pH, as well as the effect of metal ions and enzyme inhibitors on the lipase activity were also determined as described above using Tween 80 as substrate.

A correlation matrix between the esterase activities against different *p*NP substrates and the lipase activity against different Tweens was performed using IBM SPSS Statistics 25 (IBM, Armonk, NY, USA).

### 4.5. Determination of Protein Stability

Stability of LysB-His_6_ enzymes was determined using Nano-Differential Scanning Fluorimetry (DSF) on a Prometheus NT.48 instrument (NanoTemper Technologies, GmbH, Germany). Ten microliter samples of 2 μM LysB-His_6_ enzymes in 50 mM Tris-HCl buffer (pH 8) were loaded into UV capillaries (NanoTemper Technologies), and subjected to increasing temperature from 20 to 95 °C with a temperature gradient of 1 °C/min. Protein unfolding was detected by tracking changes in the intrinsic tryptophan/tyrosine fluorescence (350 nm/330 nm fluorescence ratio) as a function of temperature increase. Data was analyzed using the ThermControl software V 2.0.4 (NanoTemper Technologies) and melting temperature (T_m_) was calculated.

### 4.6. Hydrolysis of M. smegmatis mAGP

#### 4.6.1. Preparation of Mycolylarabinogalactan-Peptidoglycan (mAGP) Substrate

mAGP was extracted from *M. smegmatis* according to the method described earlier [33] with modification. Briefly, *M*. *smegmatis* cells were grown overnight in 1L of LB medium supplemented with 0.05% Tween 80 at 37 °C and 200 rpm. The cells were harvested by centrifugation at 3900× *g* and 4 °C for 20 min and washed three times with phosphate buffered saline (PBS) pH 7.4. The extractable lipids of the cell pellet were extracted with a 2:1 mixture of chloroform/methanol (15 mL per g cell pellet) at 55 °C overnight, followed by 3 successive washing steps with MilliQ quality water. Subsequently, the pellet was resuspended in 30 mL of 2% SDS and stirred gently overnight at room temperature and then collected by centrifugation (3900× *g*, 4 °C, 20 min), resuspended again in 30 mL of 2% SDS and refluxed for 1 h. SDS was removed through five successive washings (with MilliQ quality water)/centrifugation steps. The pellet was then washed with 30 mL of 80% acetone followed by 30 mL of diethyl ether, 30 mL of 80% acetone, and twice with water at the end. The resulting mAGP was lyophilized and stored at 4 °C for further experiments.

#### 4.6.2. Treatment of mAGP with LysB-His_6_ Enzymes

The purified mAGP (1 mg) was suspended in 1 mL PBS pH 7.4 containing 0.2% Triton X-100 through sonication (2 × 60 s, cycle 0.5), followed by addition of LysB-His_6_ (100 µg), and incubation for 24 h at room temperature with shaking at 300 rpm. The liberated mycolic acid was extracted with 3 mL of diethyl ether and washed with water. The organic phase (upper layer) was separated from the aqueous phase by centrifugation (3900× *g*, 4 °C, 5 min), collected and air dried. After drying, the samples were dissolved in 1 mL of organic solvent (80% n-propanol, 20% hexane) and analyzed by liquid chromatography–mass spectrometry (LC-MS) using an Accela 600 high-pressure liquid chromatograph (HPLC) coupled to an LTQ-Orbitrap hybrid mass spectrometer (Thermo Scientific). The LC was equipped with a C18 reversed-phase column (Phenomenex Kinetex XB-C18 50 × 2.1 mm, 1.7 µm particle size) and elution was performed with a mobile phase comprising 40% solvent A (100% methanol) and 60% solvent B (80% n-propanol and 20% hexane) at a flow rate of 0.2 mL/min over 10 min. The MS was operated in electrospray ionization mode with detection of negatively charged ions in the m/z range 500–1500 Da at a resolution of ∼100,000. Negative control (buffer replacing the LysB-His_6_ enzymes) and a control using *Rhizopus oryzae* lipase were included in the assay.

### 4.7. Antibacterial Activity of LysB-His_6_ Enzymes Against M. smegmatis

#### 4.7.1. In Vitro Antibacterial Activity Testing Using Different Culture Media

*M. smegmatis* was cultivated in 7H9 medium supplied with ADC, 0.2% glycerol and 0.05% Tween 80 at 37 °C, 200 rpm until mid-log phase. The cells were incubated with different concentrations (20–200 µg/mL) of LysB-His_6_ enzymes and *Rhizopus oryzae* lipase, respectively, in a microtiter plate in a final reaction volume of 200 µL; 7H9 medium without any enzyme served as negative control. The microtiter plates were incubated at 37 °C for 24 h and examined for growth. For viable counting plating assay, 100 µL aliquot from each well was diluted in PBS containing 0.05% Tween 80 and plated on 7H10 agar supplemented with OADC (Oleic acid-Albumin-Dextrose Complex), 0.5% glycerol and 0.05% Tween 80. The plates were incubated at 37 °C for 48 h followed by colony counting. Since 7H9 medium contains mono- and divalent cations (Na^+^, K^+^, Mg^2+^, Zn^2+^, and Ca^2+^), we noticed formation of a precipitate upon addition of LysB-His_6_ enzymes which might be due to formation of oleic acid salts upon hydrolysis of Tween 80 by LysB-His_6_ enzymes. Also, adding LysB-His_6_ enzymes to Tween 80 free 7H9 medium resulted in a faint turbidity, so we decided to exclude 7H9 medium when testing the antibacterial activity of LysB-His_6_ enzymes. Instead, Mueller-Hinton broth (MHB), Cation Adjusted Mueller-Hinton Broth (CAMHB), and LB culture media, respectively, were tested.

#### 4.7.2. In Vitro Testing of the Combined Effect of LysB-His_6_ Enzymes with Anti-TB Drugs

The following antibiotics were tested: Rifampicin (RIF), Isoniazid (INH), Ciprofloxacin (CIP), and Pyrazinamide (PYZ). Firstly, the minimum inhibitory concentrations (MIC) and the minimum bactericidal concentrations (MBC) of those antibiotics were determined. For MIC, antibiotics stock solutions were serially diluted (2-fold) in MHB and mixed with *M. smegmatis* (1 × 10^6^ CFU/mL) in a total volume of 200 µL, the reaction mixture was incubated at 37 °C for 24 h and examined for bacterial growth. MIC was defined as the lowest antibiotic concentration of the antibiotic that inhibited the growth of *M. smegmatis* (no visible growth at the end of the experiment).

MBC was evaluated by plating 100 μL suspension from the wells with no visible growth on 7H10 agar medium and incubating at 37 °C for 48 h and checking for growth. MBC was defined as the lowest antibiotic concentration that showed no growth. Positive controls (100 μL of bacterial culture + 100 μL MHB) and negative controls (MHB mixed with antibiotics) were included. The experiment was performed in triplicates independently. To test the combined effect of LysB-His_6_ enzymes with anti-TB drugs, modified checkerboard technique was applied [69]. Briefly, in a microtiter plate, antibiotics (half the MIC values) were diluted two-fold vertically (columns) in MHB, while LysB-His_6_ enzymes were diluted two-fold horizontally (rows). *M. smegmatis* cells were added to the wells to a final volume of 200 µL to give (1 × 10^6^ CFU/mL). The plates were incubated at 37 °C for 24 h and examined for growth. The combination effect is synergistic when the effect of the combined drugs is significantly greater than the effects of each drug individually, antagonistic when the combination effect is lower than the effect of each drug individually or additive when the effect is equal to the sum of the separate effect of each drug [70].

#### 4.7.3. In Vitro Testing the Combined Effect of LysB-His_6_ Enzymes with Cationic Antimicrobial Polypeptides (Colistin and Protamine Sulfate)

The MIC and MBC values for Colistin (Col) and Protamine sulfate (Prot) were determined as described earlier in CAMHB medium, and modified checkerboard technique was performed to determine the combinatorial effect of LysB-His_6_ enzymes with Col and Prot as described above. *M. smegmatis* cells incubated with half MIC values of Colistin and Protamine sulfate were included as controls.

## 5. Conclusions

Our study clearly demonstrates that LysB enzymes are lipolytic enzymes (combining features of esterases and lipases) that hydrolyse a range of fatty substrates besides the mAGP complex, their natural substrate in the mycobacterial cell envelope. When applied externally to *M. smegmatis* cells, LysB-His_6_ enzymes showed marginal antimycobacterial activity. However, combining outer membrane permealizers with LysB-His_6_ enzymes enhanced the antimycobacterial activity. The potential application of LysB enzymes to disrupt and kill the mycobacterial cells is currently being investigated in combination with both LysA and antimicrobial peptides.

## Figures and Tables

**Table 1:** Kinetic parameters of Lysin B (LysB)-His_6_ enzymes against *para*-nitrophenyl substrates with different carbon chain lengths.

LysB-	*p*NPB (C4)	*p*NPO (C8)	*p*NPL (C12)	*p*NPM (C14)	*p*NPP (C16)
K_m_ (µM)					
D29	422.6 ± 29.4	42.3 ± 0.445	19.6 ± 3.094	24.08 ± 0.505	37.7 ± 7.134
Omega	618.8 ± 24.98	193.3 ± 2.258	280.8 ± 3.26	98.72 ± 5.292	24.4 ± 3.116
Saal	4172.12 ± 20.104	1519.25 ± 38.596	1476.39 ± 33.184	956.7 ± 12.93	2833.32 ± 40.824
Obama12	1268.5 ± 60.032	2666.8 ± 46.54	3000 ± 20.104	1266.63 ± 86.40	800 ± 22.05
V_max_ (U. mg^−1^)					
D29	122.3 ± 2.87	9.85 ± 0.032	7.55 ± 0.039	3 ± 0.0244	2.73 ± 0.0690
Omega	111.8 ± 0.447	15.7 ± 2.35	79.8 ± 0.484	22.3 ± 0.0869	1.45 ± 0.0262
Saal	1.49 ± 0.559	0.247 ± 0.0417	0.425 ± 0.0164	0.414 ± 0.009	0.4 ± 0.0083
Obama12	0.470 ± 0.108	0.165 ± 0.017	0.17 ± 0.077	0.119 ± 0.0253	0.084 ± 0.036
k_cat_ (min^−1^)					
D29	716.5 ± 11.23	57.68 ± 12.81	44.28 ± 1.562	17.63 ± 0.955	14.53 ± 1.008
Omega	704.11 ± 18.78	452.51 ± 9.89	503.84 ± 20.33	140.78 ± 4.708	9.13 ± 1.104
Saal	11204.83 ± 38.063	1855.22 ± 39.202	6373.97 ± 54.024	3107.73 ± 12.322	3364.4 ± 56.520
Obama12	3461 ± 72.628	1215.69 ± 47.615	1256.84 ± 52.137	814.35 ± 5.129	622.07 ± 24.341
k_cat_/K_m_ (µM^−1^ min^−1^)					
D29	1.7 ± 0.368	1.36 ± 0.0434	2.26 ± 0.362	0.732 ± 0.0307	0.38 ± 0.0470
Omega	1.14 ± 0.044	2.34 ± 0.364	2.41 ± 0.1356	1.43 ± 0.047	0.374 ± 0.297
Saal	2.68 ± 0.458	1.22 ± 0.0138	4.31 ± 0.201	3.24 ± 0.211	1.18 ± 0.145
Obama12	2.72 ± 0.819	0.455 ± 0.0236	0.42 ± 0.0173	0.642 ± 0.187	0.77 ± 0.030

**Table 2:** Correlation matrix for the esterase and lipase activity data sets against *p*-nitrophenyl esters and Tweens, respectively.

	Tween 20	Tween 40	Tween 60	Tween 80	*p*NPB	*p*NPO	*p*NPL	*p*NPM	*p*NPP
Tween 20	1	0.615	0.998	0.992	0.779	0.490	−0.037	−0.004	0.953
Tween 40	0.615	1	0.661	0.528	0.973	0.988	0.765	0.785	0.804
Tween 60	0.998	0.661	1	0.985	0.815	0.541	0.023	0.056	0.966
Tween 80	0.992	0.528	0.985	1	0.706	0.393	−0.143	−0.111	0.908
*p*NPB	0.779	0.973	0.815	0.706	1	0.928	0.597	0.623	0.920
*p*NPO	0.490	0.988	0.541	0.393	0.928	1	0.853	0.870	0.712
*p*NPL	−0.037	0.765	0.023	−0.143	0.597	0.853	1	0.999	0.245
*p*NPM	−0.004	0.785	0.056	−0.111	0.623	0.870	0.999	1	0.277
*p*NPP	0.953	0.804	0.966	0.908	0.920	0.712	0.245	0.277	1

**Table 3:** Effect of different additives including metal ions, EDTA and phenylmethane sulfonyl fluoride (PMSF) on the esterase and lipase activities of LysB-His_6_ enzymes against 1 mM *p*NPB and 0.33% Tween 80, respectively.

	D29	Saal	Omega	Obama12	D29	Saal	Omega	Obama12
	Esterase Activity Against 1 mM *p*NPB				Lipase Activity Against 0.33% Tween 80			
MnCl_2_	202.7 ± 0.69	199 ± 0.22	153.16 ± 0.48	61.5 ± 0.75	106.7 ± 0.125	92.4 ± 0.08	225 ± 0.036	214.3 ± 0.079
CaCl_2_	115.25 ± 0.007	407.7 ± 0.032	90.18 ± 0.068	78.45 ± 0.001	32.2 ± 0.373	118.71 ± 0.104	137.5 ± 0.057	140 ± 0.22
MgCl_2_	145.5 ± 0.005	74.3 ± 2.07	44.28 ± 0.012	68.65 ± 0.021	46.95 ± 0.05	109.35 ± 0.14	175 ± 0.87	125.7 ± 0.22
ZnCl_2_	31.15 ± 0.96	12.23 ± 0.86	52.35 ± 1.36	38.65 ± 2.16	0.615 ± 0.07	0	300 ± 2.13	48.57 ± 0.29
CuCl_2_	22.35 ± 0.087	15.64 ± 0.42	34.21 ± 0.623	27.54 ± 0.951	5.22 ± 0.98	6.84 ± 1.13	24.67 ± 2.13	14.8 ± 3.02
KCl	218.62 ± 3.04	16.44 ± 0.56	45.08 ± 3.03	81.6 ± 2.15	93.94 ± 4.11	25.14 ± 3.02	212.5 ± 6.08	125.7 ± 7.01
NaCl	110.77 ± 2.07	70.81 ± 0.036	92.86 ± 4.03	85.57 ± 1.07	89.41 ± 0.141	103.5 ± 0.183	175 ± 0.88	108.57 ± 0.91
EDTA	87.53 ± 4.71	67.93 ± 1.09	72.95 ± 0.111	111.3 ± 4.93	88.31 ± 0.147	256.14 ± 3.09	187.5 ± 8.05	105.7 ± 6.07
PMSF	4.95 ± 0.04	6.15 ± 0.02	0	4.798 ± 0.053	12.95 ± 0.327	0	3.12 ± 0.009	8.57 ± 0.804

Activity is expressed as % relative activity. The 100% activity against 1 mM pNPB corresponds to 10, 9.77, 0.0524 and 0.0145 U/mg for LysB-D29, -Omega, -Saal and -Obama12, respectively. The 100% activity against 0.33% Tween 80 corresponds to 42.1, 1.058, 7.11 and 4.49 kU/mg for LysB-D29, -Omega, -Saal and -Obama12, respectively.

**Table 4:** Log_10_ reduction of *M. smegmatis* after treatment with 100 µg/mL of LysB-His_6_ enzymes alone and in combination with Colistin (1 µg/mL) and Protamine sulfate (10 µg/mL), respectively in Cation Adjusted Mueller–Hinton Broth (CAMHB), NA: not available.

	Alone	Log_10_ Reduction Plus 1 µg/mL Colistin	Plus 10 µg/mL Protamine Sulfate
LysB-D29	1.1 ± 0.062	3 ± 0.073	1.8 ± 0.086
LysB-Omega	1.32 ± 0.048	3.45 ± 0.066	2.1 ± 0.095
LysB-Saal	1.44 ± 0.052	3.1 ± 0.048	1.9 ± 0.056
LysB-Obama12	1.36 ± 0.06	4 ± 0.065	2.8 ± 0.091
Colistin	0	NA	NA
Protamine sulfate	0	NA	NA

**Table 5:** Nano-differential scanning fluorimetry (DSF) melting temperature for LysB-His_6_ enzymes.

LysB-	T_m_ (°C)
D29	54.7 ± 0.17
Omega	57.7 ± 0.3
Saal	45.7 ± 0.1
Obama12	47.9 ± 0.5

## Supplementary Materials

The following are available online at https://www.mdpi.com/1422-0067/21/9/3176/s1. Figure S1: SDS-PAGE of purified LysB-His_6_ enzymes. L1 Precision Plus Protein^TM^ All Blue Prestained Standards (Bio-Rad), L2: LysB-D29 (MW: 29.3 kDa), L3: LysB-Omega (MW: 31.5 kDa), L4: LysB-Saal (MW: 37.4 kDa), L5: LysB-Obama12 (MW: 36.7 kDa). Figure S2. Nucleotide sequences of the codon optimized genes used in the current study. Figure S3: 3D conformations of poses of *p*NP ligands upon docking to LysB proteins. Each diagram illustrates (in order) the overall surface of protein with its ligand, the shape of active site with the docked ligand, the interactions of ligand atoms with different residues of the corresponding protein. Hydrophilic residues (pink color), hydrophobic residues (green color), *p*NP ligands (black color). Orientation pose: ligand-protein conformation where the catalytic Ser faces ligand’s ester bond (C=O) with no H-bond formation. NDP: (No Detected Pose) neither binding nor orientation pose were detected. Stars indicate catalytic triad residues of LysB-D29. Table S1: Minimum Inhibitory Concentration (MIC) and Minimum Bactericidal Concentration (MBC) values of the antibiotics used in the antibacterial activity assay against *M. smegmatis.*
