# Supplementary material for: Exploring the Enzymatic and Antibacterial Activities of Novel Mycobacteriophage Lysin B Enzymes
Source: Int J Mol Sci. 2020 Apr 30;21(9):3176. doi: 10.3390/ijms21093176 (PMC7246905; doi:10.3390/ijms21093176)
Supplement: Supplementary file 1 [file ijms-21-03176-s001.pdf]

Supplementary information

Exploring the enzymatic and antibacterial activities of novel Mycobacteriophage Lysin B enzymes

Adel Abouhmad[1,2], Ahmed H. Korany[3], Carl Grey[1], Tarek Dishisha[4] and Rajni Hatti–Kaul[1*]

[1]Biotechnology, Department of Chemistry, Center for Chemistry and Chemical Engineering, Lund University, P.O. Box 124, SE–221 00 Lund, Sweden; adel.attia@biotek.lu.se; carl.grey@biotek.lu.se
[2]Department of Microbiology and Immunology, Faculty of Pharmacy, Al–Azhar University, 715 24 Assiut, Egypt
[3]Department of Microbiology and Immunology, Faculty of Pharmacy, 62513 Nahda University, Beni–Suef, Egypt; ahmed.hassan@nub.edu.eg
[4]Department of Microbiology and Immunology, Faculty of Pharmacy, Beni–Suef University, 625 11 Beni–Suef, Egypt; Tarek.Dishisha@pharm.bsu.edu.eg

*Correspondence: Rajni.Hatti–Kaul@biotek.lu.se; Tel: +46-46-222 4840

Table S1: Minimum Inhibitory Concentration (MIC) and Minimum Bactericidal Concentration (MBC) values of the antibiotics used in the antibacterial activity assay against *M. smegmatis.*

Antibiotic	MIC	MBC
**Rifampicin**	0.6 µg/ml	1.2 µg/ml
**Isoniazid**	58.59–234.375 µg /ml	500 µg /ml
**Ciprofloxacin**	0.005–0.01 µg/ml	0.01–0.02 µg/ml
**Pyrazinamide**	7.5 mg/ml	13.5 – >13.5 mg/ml
**Colistin**	2 µg/ml	2 µg/ml
**Protamine sulfate**	20 µg/ml	20 µg/ml

Figure S1

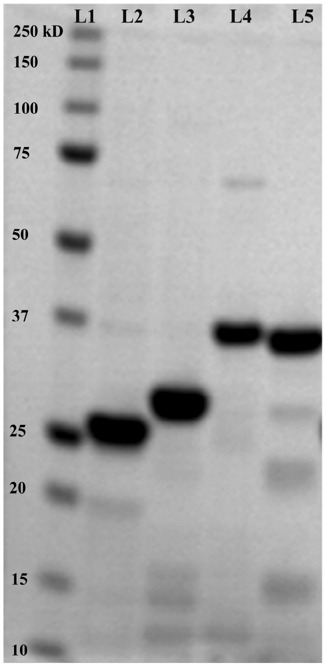

Figure S1. SDS–PAGE of purified LysB–His_6 enzymes. L1 Precision Plus Protein™ All Blue Prestained Standards (Bio–Rad), L2: LysB–D29 (MW: 29.3 kDa), L3: LysB–Omega (MW: 31.5 kDa), L4: LysB–Saal (MW: 37.4 kDa), L5: LysB–Obama12 (MW: 36.7 kDa).

Figure S2. Nucleotide sequences of the codon optimized genes used in the current study

- **LysB–D29 (Uniport ID: O64205)**

ATGAGCAAGCCCTGGCTGTTCACCGTTCACGGCACGGGCCAGCCCGATCCCCTCGGGCCTGGCCTGCC
TGCCGATACGGCACGCGACGTACTTGACATCTACCGGTGGCAGCCCATCGGCAACTACCCCGCTGCGG
CCTTCCCGATGTGGCCGTCGGTCGAGAAGGGTGTCGCCGAGCTGATCCTGCAGATCGAGCTGAAGCTG
GACGCGGACCCCTACGCGGACTTCGCGATGGCGGGTTACTCGCAGGGAGCCATCGTGGTTGGCCAGGT
GCTCAAGCACCACATCCTGCCTCCGACGGGCAGGCTCCACAGGTTCCTGCACCGGCTCAAGAAGGTCA
TCTTCTGGGGTAATCCCATGCGGCAGAAGGGCTTTGCCCACTCTGACGAGTGGATCCACCCGGTCGCT
GCCCCTGACACCCTCGGAATCCTCGAGGACCGGCTCGAAAACCTGGAGCAGTACGGCTTCGAGGTCCG
CGACTACGCCCACGACGGTGACATGTACGCCTCCATCAAAGAGGACGACCTGCACGAATACGAGGTCG
CCATCGGCCGGATCGTGATGAAGGCCAGCGGCTTCATCGGTGGCCGGGACTCCGTGGTAGCCCAGCTC
ATCGAGCTTGGCCAGCGTCCGATCACCGAGGGAATTGCGTTGGCGGGAGCCATCATCGACGCCCTCAC
GTTCTTCGCCCGCTCTCGTATGGGCGACAAGTGGCCGCACCTCTACAACCGCTACCCGGCGGTCGAGT
TCCTACGACAGATCCACCACCACCACCACCACTGA

- **LysB–DS6A (Uniprot ID: G8I4E1)**

ATGACCTGGATTGGCTGGCAGCAAGGTATGGCGGGAGAGCCGGTAGCCGCTGCGAAGCGTGAGCTTCG
TCGCAAATTCAGTTATGCTAAACATCTGGATGACAGCGACGTATTTGATCTGGAGTTATTGGCCGTTT
TGATCGAGTACCAACAAAAAAAGAACGCGACTGGTTATACACCTCGCCTGCGCGAAGACGGCGTTCTT
GATTGGGCCACTCAAGTTGCGATTGGTACGGTGCAAACTGGGCCCCCTCCTAAGGCAGGCACGTTATT
TACTGTGCAAGGAACTGGCGTTGATATGTGGACAGGACCCCCGGCGGACACGGCTCGTGCCGTGGAGG
GCGACTGGGAATGGCAGCCGATTGGCAATTACCCTGCTTCACCGTTCCCAATGTGGGCTTCCATCTGG
CAGGGGATTGAAGAACTGCGCTTTCAGATTCGTCGCCATGCTACGGAAAAACCGGGGAAAGCCATTGC
CCTGGCAGGTTTCTCACAGGGTGCCGTAGTCGTAAGTTGGGTCTACAAGTGGGACATTGCACTGCCCG
GTGGCATGCTGCACGACTTGCTGCCACAGGTGGCAGCGGGTGTAACGTGGGGTAATCCCATGGCAGAA
AAAGGCAAGTTTCATGGTAACCGCTTCGCCGGCTGGCCTGTGCCTGCCGGAATGGGGATCAACCGTGA
CCGCTTGGAAAATACCCCCGCATTATGGCTTGACTTTGGGCACGGTCGCAATTCACCTTGGGGCCAAG
ATATTTATTGCGACCGCCCGGATAATTTGGCTGGTCAGGACATGGAGCTTATTTGTGACCTGGTCATG
GCTCAGGACCTGTGGCGCTTTGTGACAGACTTCGCAGTAAAAGCTGGGAACGTGATGGCGTCGCCTGA
GACGGAGTTACCCGCAATCTTGGATGCAGTTACACAAGCTGGTATGTTTTTCATTGTTAATCGTACTG
CCCCGCATCTGACTTATGACGTAGGTCCTGCCATCGATTACCTTCGCCAAGTTGCGCGCGACCGCCGC
TTGGCTTTAGCCGCGCATCACCATCATCATCACTAA

- **LysB–Omega (Uniprot ID: Q854L1)**

ATGTTGAACGGTGAATTTTACGTTCCGGCAGGGACAATCATTACTGCCAACGGGACAGGGGTTCCAGA
TGGTGCGGGGCCCGGATTTGGTGCCGATCTGGCTCGCGAACTGGAGCGTGTGGCTGAAGGCATGTGGA
TTTGGAAATGGATCAACTACCCAGCAGCAGTAATGCCGATGCGTCCGAGCATTAATACGTTGCGTTTG
GCTTTAAAAGAGATGATCCGTACCACTCCGGGCAAAATTGTTTTATCGGCGTACTCTCAATCGGCCGT
GGCTTTTGCTTACGTATGGCGCGACGACATTCTTAACCCCGATGGTGAACTTCACGACCGCCTGGATG
ATATTGTAGCAGTAATCTTGTACGGCAACCCAGTTCGCGCCCCTGGGATTGCTTATGGCAACGAGTTA
GGCGGTCAATCTGCCCCCGGCAAGCTGAATGGGCATGTCACCGGCGGCATCGCCGGGCCTGATTGTTT
GCGCCCGGAAGAGTGCGTCCATCCCGTCACTGGGCGTCGCATTGTACTGGACTTCGCTAATGCCGGTG
ATTTGTACGCTGCTGCACCCGTTGGCGCGGAACCCTGGGTCAAAGAGACTGAGGTAGGCCATAACGAG
ACGCTTATTTACGAAGCTGTTATGGATTTTAATGGCCGCGATATCCTTGCATTCGCCAAAGAGATCGC
ACAGATTCTTACAATGCCCTTGAGCCAGGTCGTACCGCTGGTACAAGCAATTATCAACGGGTTAAGCT

TTCTTGTCCAGGGCCCGAAAGCTCCGCACTGGACGTACACGATTGGCCCGGCAGTCGATTACTTAGTC
CGCACGGGCAATGAACTTCGTAAAAATCATCACCATCATCATCACTAA

- **LysB–Saal (Uniprot ID:W6AV51)**

ATGCGCATCGACGGTCAATATGTGGGACTGGGGCTTGGGGATTCTTCAGACGAGATCCGTAAAATCAA
GGCGTTCATGCGCCGCAAATTCGCGAGCTATGCCGGAGATTTAGCGGATACTCCTTTGTACGATGAAG
CTATGACCGCAGCAGTGGCCGAGATGCAAGCGCGCTACAGTGCGGCTGGTCAACTTCGTGCTGGATTG
TACATTCCCGGTATCGTAGGAGCCGAAACTAAGTATGTGATGGGTTACTTACCCCGCCCCGTCGTTGA
TACTCGTCCGGTACTGATCACTGTGTGTGGTACGGGTGTTCCATGGTGGATTGGGCCTGATGCAGATA
CTGCTCGCGCTGTGGAGGATAAGTACCTGTGGCAACCGATCGGTTATCCTGCAGCCCCTTTTCCAATG
GGAAAATCCATCGCGGCGGCTATTACCGAGACTCACAACCAAGCCAATCGCTGGCGCGAGCGCATCGA
AACTCACGGCGCGGCCTTGGCTGGCTATAGCCAGGGTGCAGTGGTAGTGTCAGAGCTTTGGATGAACC
ACATTGCGCCGGAGGATGGTTCCCTTCATTGGATGAAACCACACATTGAAAAAGCCGTCACTTGGGGG
AACCCAAATCGCGAGTTGGGGCACGTATGGGCGGACCACGGGGGTTCTCCTCGTGCGCCGTCTAATAC
CCAAGGCGTTTCAAGTAACGGAATGCGTGATACGCCCCCATGGTGGCGTGATTATGCTCACCAGGGTG
ACCTTTATGCGTGTACAGAGCCAGGGGATACTCAAGAGGTGCGTAACGCGATTTGGCAAATTGTACGC
GACTTGGATTTATTTACAGGGCCCGACAGTTTGTTAGCCCAGGTTGTAGAGTTGGTGCAGGCCCCTCT
GCCCGAAGCTATCGCTATCACAAAGGCGCTTTTGGATGCCGGTATGTTCTTCGCTAAACGTACAGGCC
CACATGTGGACTATAACGTACAGCCAGCGATCGATTACCTGCGCACACATCATCATCACCATCACTAA

- **LysB–Echild (Uniprot ID: W0LJP4)**

ATGTCGTTACAACTGGGTTCAAATGGTGACCTTGTAAACCGCTGGATTCGTGTAATGAAGGCACGTTT
CGCGTCATACGCAGGTAAGTTGCGCGAAGATGCGTACTTCGGTTTGGATGACCAGGCGGTTCAACGCG
AATATGAGACCCGTACTCATCAAACACCTGATGGAATTGTCACGGACGGCGACTTGGCCTACTTACTG
CCTGCTAAGCCCTGGTTGTTTACTGTGCATGGCACCGGGATGCCGGACCCGCTGGGACCAGGGCTTCC
TGCGGATGTGGCTCGCGATGTCCTGGATATCTACCGTTGGCAGCCAATCGGGAACTACCCCGCCGCCG
CCTTCCCGATGAAGCCATCGTACGACAAAGCAATCGCAGAGTTAGTCCTGCAGATTGACTTGAAACTT
GCGGGGAATAACGACGAGTTTTCTATGGCTGGATACTCACAAGGTGCCATTGCTGTCGCGTATGTGCT
TAAACACGAAATTCTGGACCCAAAGGGGCGCTTGCACAAATATGTTAATCGCTTGAAGAAGGTCGTGA
TGTGGGGGAATCCAATGCGCCAAAAAGGATTTGCTCATTTTGATGAGTGGATCCATCCTGTAGCAGCT
CCCGATACAATGGGCATTTTGGAGGATCGCTTAGAGAATCTGGAACAGGCGATGCAACAGTATGGGTT
TGAAGTACGTGACTATGCTCATGATGGCGACATGTATGCAAGTATCAAGGAAGATGACATGCACGAGT
ACGAGGTTGCAATCGGCCGTATCGTTATGACCGTAAAGGGGTTCTACGGCGGGAAGGACTCGTTAGTC
GCGCAATTGGGTGAGTTAGCTGGCCACCCTTTGCGCGAGAGCATCGCGATGGCACGCGCGATTATTGA
CGCGATCTCGTTTCTGGCGAAAAGCACGCAGGGCGAAAAGTGGCCCCATTTGTATAATCGCTACCCCG
CTGTCGCTTTTTTGCGTCAGCCCCATCACCATCATCATCACTGA

- **LysB–Enkosi (Uniprot ID: A0A0M4R3S7)**

ATGTCTAAGCCTGTGCTGTTGACTGCTAGTGGGACGGGTGCGGATATGTGGTCTGGATATCCGGCGGA
TCTTGCGCGTCGCGTCGAGGACCTTTACTACTTTCAGCCAATCTACTATGGTCCAGGTGGGATTCCGG
CGACGGTACCAATGGGGGCGAGCGCACAGTCTGGCGTAGATATGGGAGTACGTCTGGTACTGGAGTGC
GAGGCTCGTCCGTCGCGCGACGTCCCTGATGGGTATGCCTTGTGTGGCTACTCTCAAGGGGGCATGGT
TGTATCCGCGTTGTTGGACGAGTTTCGCACGGGCCGCCTTCGCCACTTGAATGGCAAATTGATGGCGG
GCAGCACCTTTGGAAACCCGTGGCGCGAGCTTGATTCTAACGGCGGGCGCGGCATCAGCGACCGTCGC
ATTGTGGGGACCCCCGACTTCTGGGTGGATGAGTTCGACCCAGGAGACATTTATGCAAACGTGCCTAA
CAATGATGTCGGGGATGATATGACTGCCATTTTTAAGCTGGTACAGCTGCGCTCACTGGCTGACCTGA
TCGGTATTGGATCGATTATGGATCGCGTAATGGACGTTCTTCGCGGCCCATTGAGCGGGATGCGCTCC
GTAGTTGAGGCGATTGTTCGTGGCTTATTATTTTTCGGCCGTAAGCCGCCAACAGCGCCCCATACAGA
GTACCATTTGCGCGAGGTCCGTCCGGGAGTGACGTATTTTGAACATGCAGTCGCCCACATGCGCGCTA
TGGCCGCCCATCACCATCATCATCACTAA

- **LysB–Obama 12 (Uniprot ID:W0LJG8)**

  ATGCTGAAGTTAGGATCAAACGGGCTGATGGTTGCGGCGTGGACGGCAGTTATGCGTATCCGTTTCGC
  TTCGTATGCTCTTGGTGTAAACGGTTTACCGATCAAGGTAGATGGCTACTTTGGTTATGATGAAGAAA
  AAGTGCAAAAGGAGTATCAACGTCGTACAGGACAGTTCCCGTCTGGACAAGTATCGCGCGAGGATCTG
  CATCGTCTGGGTTTATTGCCAACTTTAATCAGCATTCACGGCACAGGTCAAGCAGACCCTTTTGGCAT
  CGGGTACCCGGCAGACATTGCTCGCCGCGTGCTTGATCTTTATTGGTGGCAGCCGTTAGGCAACTACC
  CGGCGAAAGCTGTCCCTATGAACGGATCGGTTGACCAGGGTGAACGTGAGGGCGTCCGTTTGATCTCT
  GACCCCCTTATCGTGCCAGGTCCAACAGCGTTTGTAGATTATTCCCAAGGTTCAGTGATTGGAGGGCG
  CTTACGCAACCGCATGCGCCGTAAGGAGCTGCGCGGAGAGCTTGTCGCCGCAGCCAGTTTTGGAAATC
  CAATGCGTCTGCGCGGCCATTACGCAGGCAACGTAGACCCTGGCGGTGAAGGTATTGATCCACGTCAA
  GAGCTGGCTGCTGAACCATTCCGCATCGAGTTAGCTGCGAAAGGCGACTTGTACACGACTTGCCCTGG
  CGGTCAGTCAGGCGAAATGGAACGCGCCATTTATCATGCGGTTTTTAGCAAGTTCATCGGAGAAGATA
  CTCTTATCGAGCAAGTATGGGAACTTGCTAAAAATCCATTTCGTGAAGTCCCGGCCGCCGTGAAGGCT
  ATTGTGCGCGGCGGAATGTTCGCGATCCGTGGCACAGGTCCTCATGTTCGCTACCATATTGATCAATG
  CCCAGGGACGGGAATGACATATTACGAATACGCGGTCAAGCATTTGCGCGACACCGCAGATGCGCGTT
  TGCGCCGCATTGTCGCGTCTGTAACACATCACCATCATCATCACTAA

- **LysB–Pumpkin (Uniprot ID: C9DCK3)**

  ATGACCACCTACGGCGAGCTTAAAGCATTGCGCCTTGGGGTCAAGTATGTACGCCATACCCTTTTCAC
  AGTGGCGGGGACTTGGGCTGATATGTGGTCAGGTTACCCAGCCGACGTTGCCCGCTTAGTTGACGAGG
  ACTTATTCCGCTGGCAACCCGTGTGGTATCCAGCCTCCTTTGGCCCGGTTGGAAACCCCTTGGGTCGC
  TCCTACCAAGAGAGTGTACAAGACGGCGTGAAAGAGTTGATCCGCTTAATTAATGCAACTCCGGGCAC
  GTTTGCCTTAGTGGGGTACAGCCAAGGCGCGGAGGTGGTCTCTCGCGTGCTGCTTGAGATCCTGTTCG
  GGAGTTTACGCCATCGCCTGAAAGACTTTATCGGAGGCGGTTGCTTCGGGAACCCCTACCGCGCCAAG
  GGAGTCTCATATCCAGGTTCTGGTTTGCCGACATCAGGACACGGAATTGCGCCTGTGAACTTAGCCCC
  CGACATCCTTCCTGCGGAGATGTGGGAGGAGTGGTGGAATGAGGGGGACTTGTATGCTCAGAACTTAG
  ACGGAAAATCGGGGGAGATTATCACCTCGTTTTACGACATCTTGACAAAGCTTCAATTCCATGACATG
  CTTGGGTTAGCCGTCAATATGTTTAAAGCACTGTCTAACGATAAGGGGATCATCGCACAAGTCATGCG
  CGTGCTGGCGGTCCCTCTGCCGGGGGTGATTGATGCGGGCCGCGCCGCCGTTTACGCTGGCACCTTCG
  CTGTCCAAGGGACCCGCCCGCACATTACCTTAGCTGAAACCGGACGTGTAGCCCGTGCTGTGTGGCAT
  TTAAATCGCATTGGTGCCAAGACATTAGCGCGCGCCTCACATCACCATCATCATCACTAA

Figure S3

A~~a~~. LysB–D29 like orientation (Tunnel conformation)

LysB–D29: Orientation pose 163, *p*NPS, S = –8.07

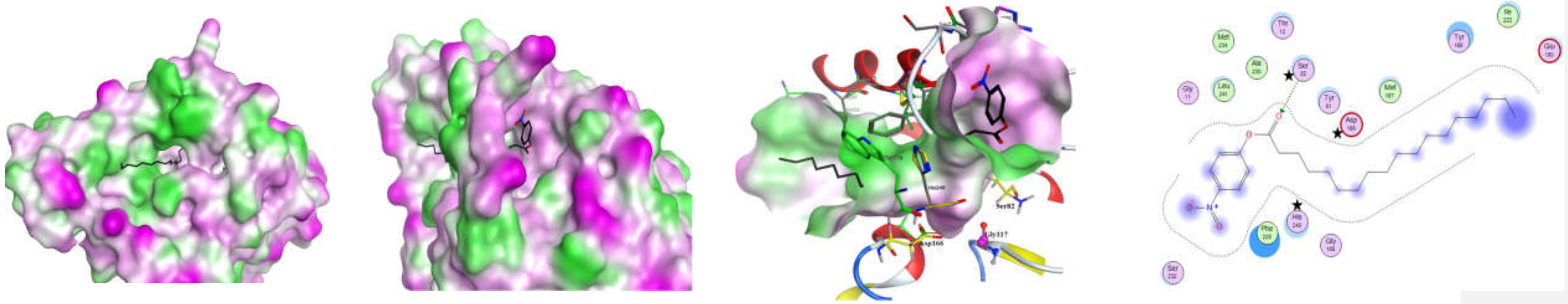

~~b.~~B. Inverted LysB–D29 orientation

Formatted: Font: (Default) Palatino Linotype, (Asian) Calibri, 12 pt, Bold

Formatted: List Paragraph, Indent: Left: 0 cm, Hanging: 0.5 cm, Add space between paragraphs of the same style, Numbered + Level: 1 + Numbering Style: A, B, C, … + Start at: 2 + Alignment: Left + Aligned at: 1.27 cm + Indent at: 1.9 cm

LysB–Obama12: Orientation pose 160, *p*NPP, S= –6.5

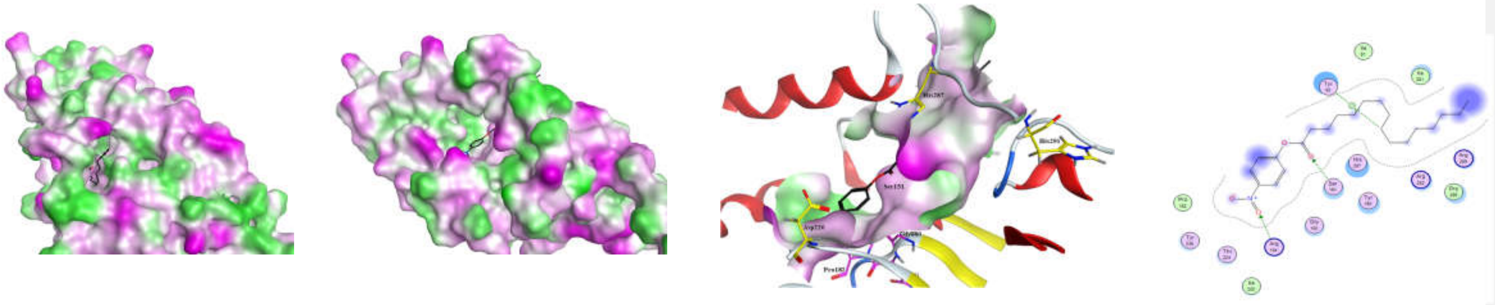

Ce. Long shallow bowl

LysB–Omega, no detectable orientation pose, *p*NPP

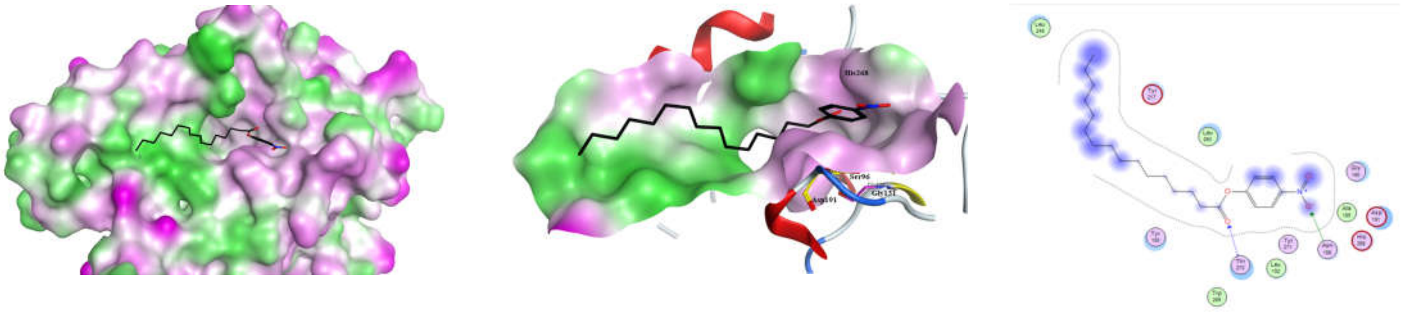

D~~d~~. Deep funnel conformation (Very deep funnel)

LysB–Saal: Orientation pose 165, *p*NPP, S = –7.5

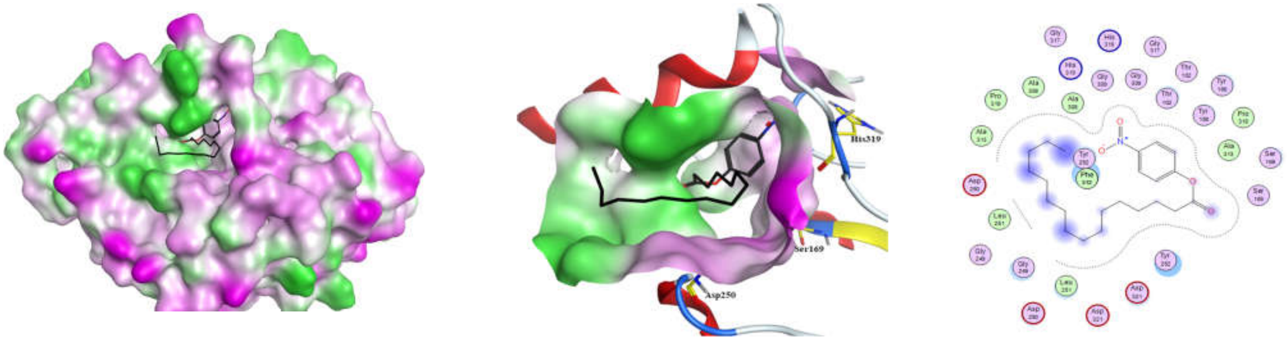

Figure S3: 3D conformations of poses of *p*NP ligands upon docking to LysB proteins. Each diagram illustrates (in order) the overall surface of protein with its ligand, the shape of active site with the docked ligand, the interactions of ligand atoms with different residues of the corresponding protein. Hydrophilic residues (pink color), hydrophobic residues (green color), *p*NP ligands (Black color). Orientation pose: ligand–protein conformation where the catalytic Ser faces ligand's ester bond (C=O) with no H–bond formation. NDP: (No Detected Pose) neither binding nor orientation pose were detected. Stars indicate catalytic triad residues of LysB–D29.
